# Craniosynostosis: Experience From a Single Tertiary Center in India

**DOI:** 10.7759/cureus.82179

**Published:** 2025-04-13

**Authors:** Manraj Singh, Mehak Nibber, Gurjinder Kaur, Uday S Raswan, Ariana Joseph, Brittany Zaita, Jake Singh, Adityabikram Singh, Akshi Talwar, Harkanwal Kaur, Deepti Taneja, Altaf U Ramzan, Sarabjit S Chhiber

**Affiliations:** 1 Basic Biomedical Sciences, Dayanand Medical College and Hospital, Ludhiana, IND; 2 Basic Biomedical Sciences, Touro College of Osteopathic Medicine, Middletown, USA; 3 Neurosurgery, Amandeep Hospital Pathankot, Pathankot, IND; 4 Urology, Lenox Hill Hospital, New York City, USA; 5 Basic Biomedical Sciences, Creighton University School of Medicine, Phoenix, USA; 6 Neurosurgery, Paras Hospitals, Srinagar, IND; 7 Neurosurgery, Sher-I-Kashmir Institute of Medical Sciences, Srinagar, IND

**Keywords:** frontal bone remodeling, metopic suture, neurosurgical intervention, non-syndromic craniosynostosis, suturectomy

## Abstract

Introduction

Premature fusion of one or more cranial sutures results in a diverse set of conditions collectively known as craniosynostosis. It is primarily responsible for cosmetic issues and occasionally associated with complications like brain growth restriction, raised intracranial pressure (ICP), and blindness. Management ranges from conservative surgical procedures such as suturectomies to more extensive procedures, including frontal bone remodeling with fronto-orbital advancement (FBR with FOA) and total calvarial reconstructions (TCVR). Currently, there is no consensus on an ideal procedure for a particular type of surgery for this condition.

Methods

A retrospective review of 26 consecutive patients treated at a single tertiary center in India was performed. Sloan's surgical outcome class and parent satisfaction score to compare different forms of intervention. Transfusion requirements, length of hospital, and increase in head circumference post-operation were also used.

Results

The mean age in our cohort was 10.9 months, with a ratio of 9:4 male-to-female. The overall assessment of pre- vs. post-operative head circumference revealed a strong significant mean improvement from 42.85 cm to 44.73 cm (p<0.001). A comparison of measured variables for all 26 patients revealed a significant difference in Sloan's surgical outcomes class (5.4 vs 1.2, p<0.001), Parent satisfaction score (5.1 vs 9.1, p<0.001) and increase in head circumference (cm) post-operation (0.74 vs 2.39, p<0.001) when comparing suturectomies vs extensive procedure like FBR with FOA and TCVR.

Conclusion

Our results favored FBR with FOA and TCVR over simple suturectomies for more satisfactory and long-lasting results with acceptable mortality and morbidity.

## Introduction

Craniosynostosis encompasses a diverse set of conditions resulting from the early fusion of one or more skull sutures. In most instances, craniosynostosis affects a single suture and does not lead to medical or neurological issues. For these cases, surgical intervention is primarily recommended for aesthetic reasons. However, when multiple sutures are involved, complications can arise, including restricted brain growth, elevated intracranial pressure, hydrocephalus, secondary Chiari malformation, and vision loss [1,2]. These complications, along with cosmetic restoration, serve as medical indications for surgical treatment in such cases [1,2].

Surgery for craniosynostosis has evolved over decades with an emphasis on early intervention, but still, there is no consensus on the ideal procedure. Most earlier reports do not quantify results that would allow objective analysis and comparisons. In developing countries like India, the cases present late and tend to have more fixed deformities [3]. We examined the results of 26 consecutive patients treated at a single tertiary care center and adopted a seven-category outcome classification system as devised by Sloan et al. [4]. Clinical outcomes and parent satisfaction scores were also used to compare the results of different forms of intervention. We hypothesize that due to late presentation, extensive procedures like frontal bone reconstruction (FBR) with fronto-orbital advancement (FOA) and total calvarial reconstruction (TCVR) will provide superior functional and aesthetic outcomes as reflected by parent satisfaction scores and Sloan surgical outcome classes, despite requiring more resources and lengthier surgeries.

Our study objectives are as follows: 1) To compare various pre-operative and post-operative variables between simple procedures (suturectomy) and extensive procedures (FBR with FOA, TCVR) in order to determine any significant difference that could affect the safety of the procedures; and 2) In late presentation of craniosynostosis (>6 months), to determine whether extensive procedure show better results than simple suturectomy in terms of post-operative head circumference change, Sloan Surgical Outcome Class and parent satisfaction score.

## Materials and methods

Study design

A retrospective review was performed for all patients (n=26) who underwent surgical correction of craniosynostosis at a tertiary care center in India from August 2005 to July 2021. Patients were classified based on the type of synostosis present: sagittal (scaphocephaly), metopic (trigonocephaly), unilateral coronal (anterior plagiocephaly), bilateral coronal (brachycephaly), and multiple sutures (multiple/pansynostosis). Patients with non-syndromic craniosynostosis were included in the study. Syndromic craniosynostosis and positional plagiocephalies were excluded from this series. Parental informed consent was obtained for the present study, and the medical records of individuals reported within this cohort were retrieved and reviewed. This retrospective study was performed in accordance with clinical study guidelines in India and was reviewed and approved by a local ethics committee.

The patients were divided into two groups depending on the extent of the surgical procedure: group 1 (less extensive procedures, including suturectomies) and group 2 (more extensive procedures, including FBR with FOA and TCVR).

Age at diagnosis (months old), preoperative hemoglobin level, transfusion requirement (mL), length of hospital stay (days), and operative time (hours), pre- and post- operative head circumference (cm), increase in head circumference following surgical intervention (cm), parent satisfaction score, Sloan's surgical outcome score were recorded and evaluated for statistical significance.

Determination of synostosis type

Determination of synostosis type was performed by clinical examination, as well as supportive brain imaging: preoperative CT head was used to delineate type of suture involvement, and MRI brain was performed to rule out any structural brain abnormalities. Synostosis classifications depend on the type of suture affected and include sagittal (scaphocephaly), metopic (trigonocephaly), unilateral coronal (anterior plagiocephaly), bilateral coronal (brachycephaly), or multiple sutures (pansynostosis). Patients with clinical NSC were included, whereas syndromic craniosynostosis and skull deformation due to positional plagiocephaly were excluded from this series.

Operative technique

**Figure 1:**
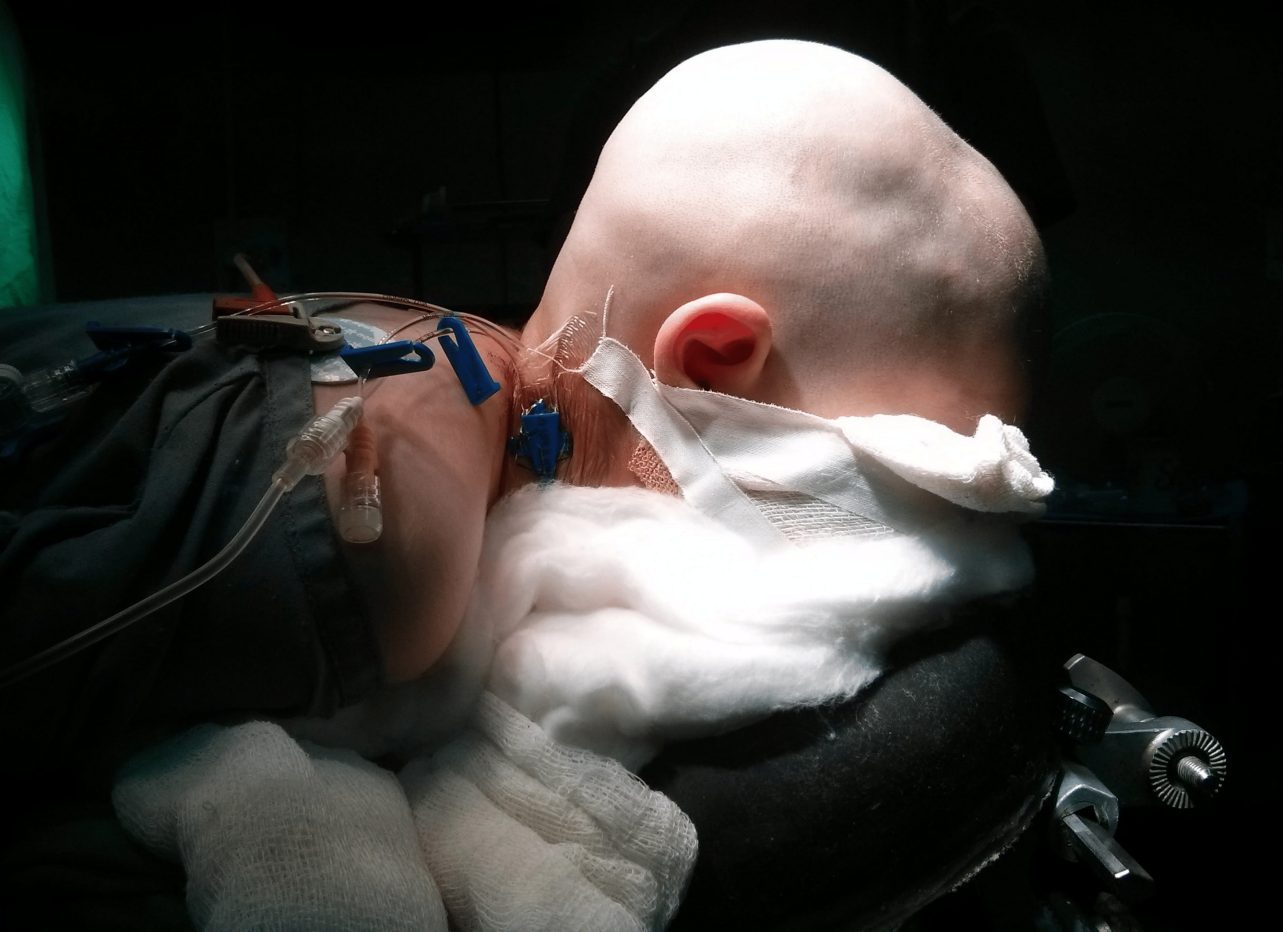
Supine neutral position for FBR with FOA FBR with FOA - frontal bone remodeling with fronto-orbital advancement

The operative procedures used were based on the technique earlier described by senior authors and can be divided into the following parts depending on the tailored needs of a particular clinical situation [5]. Patients were placed into the prone sphinx position for TCVR and supine neutral for FBR with FOA (Figure 1). A skin incision was created in a zig-zag fashion coronally from ear to ear.

Frontal bone remodeling with fronto-orbital advancement(FBR with FOA)/ anterior cranial vault reconstruction

**Figure 2:**
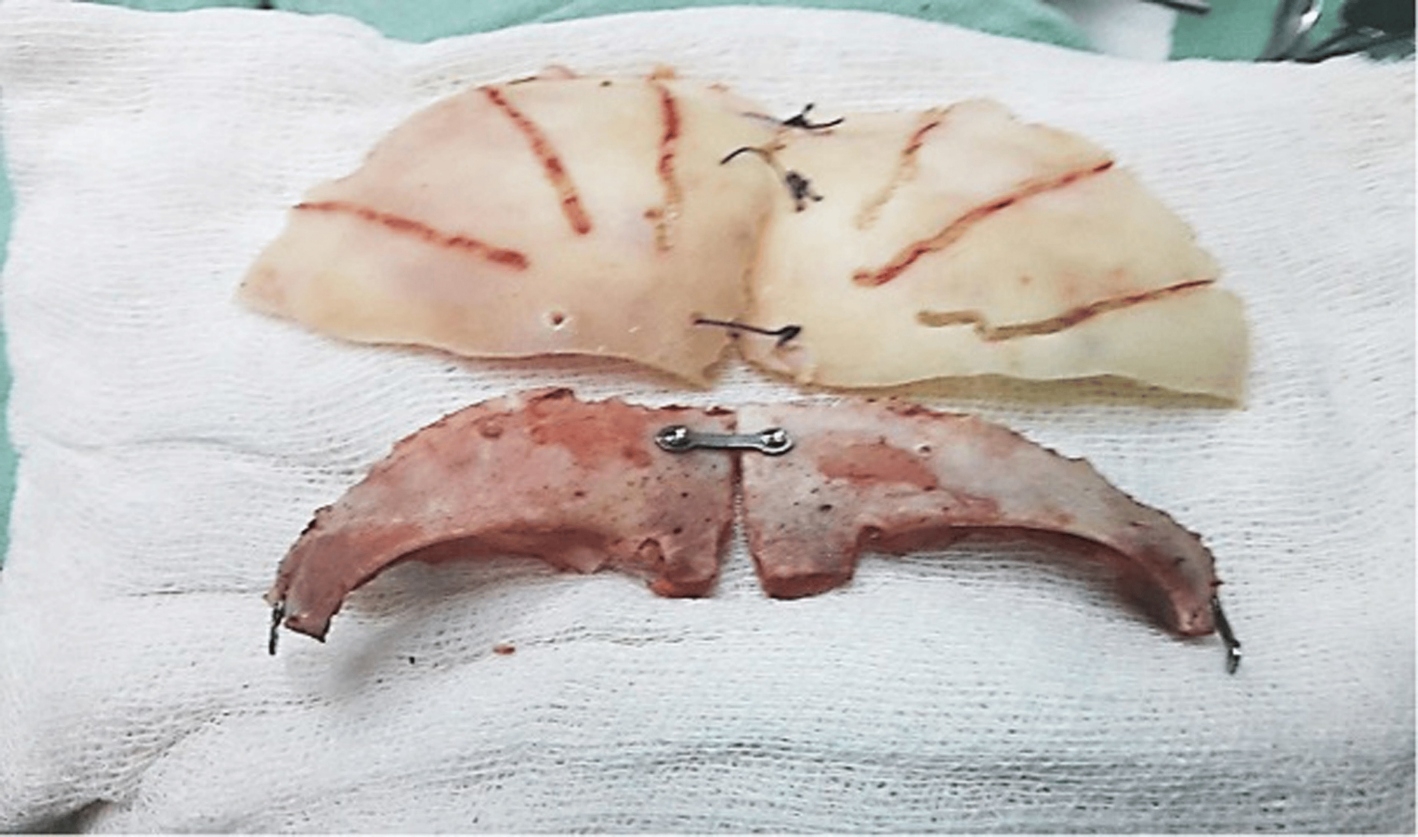
Creation of Bone Flaps for FBR with FOA FBR with FOA - frontal bone remodeling with fronto-orbital advancement

**Figure 3:**
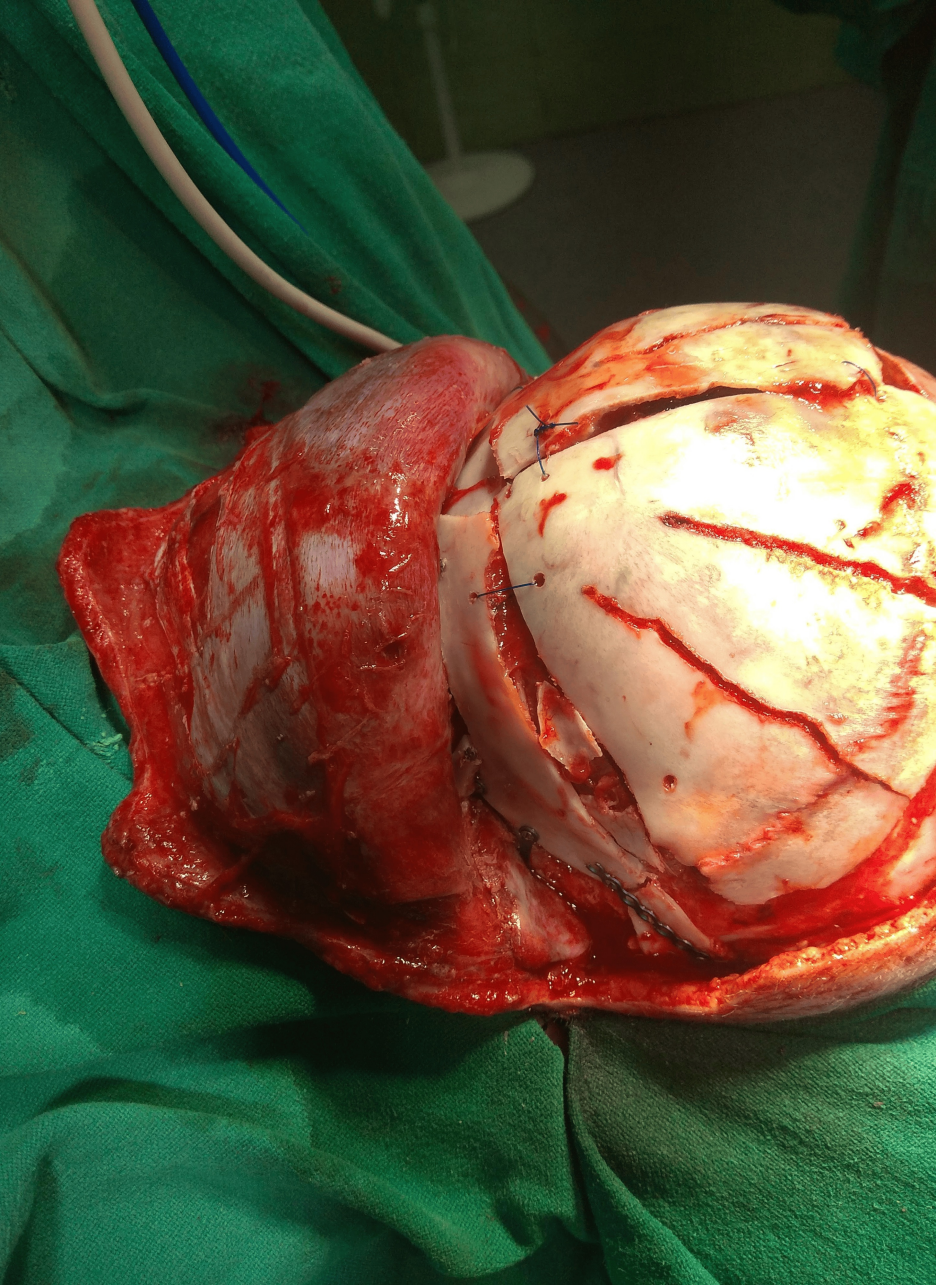
Remodeling of supra-orbital bandeau in FBR with FOA FBR with FOA - frontal bone remodeling with fronto-orbital advancement

After frontal craniotomy, just behind the coronal sutures and 2cm above the supra-orbital ridge, the harvested bone flap is divided in the center, and the barrel-stave osteotomy (BSO) is created. These bone flaps are then gyrated in such a way that the inferior bone margin lies medially and the bone adjacent to the metopic sutures faces the supra-orbital (SO) bandeau. The two flaps are hitched together loosely in the middle with prolene sutures, thus creating a floating forehead flap [5]. The supra-orbital bandeau (SOB) is freed by a lateral osteotomy of the lateral orbital rims just beneath the fronto-zygomatic suture. Then an inferior osteotomy at the nasion just above the canthal ligament insertion and a final cut are made in the anterior cranial fossa; first in the median plane just ventral to crista galli with extension laterally along the orbital roof in the direction of key burr hole bilaterally, connecting it with lateral orbitotomies, thus detaching the SOB form skull base [5]. A BSO is created in the part of the orbital roof left behind and, in the part, attached to the bandeau. This helps to increase the orbital volume, along with SO advancement. In some cases, because of angulation in the midline, the osteotomy is fashioned in the midline and again secured with a mini-plate in the front to create the required angle, making it nearly flat (Figure 2) [5]. The superior edge is attached loosely to the forehead flap with prolene sutures, thus generating an anterior flap. The lateral orbital margins are laid down just anterior to cut the inferior margins of the orbital rim. They are secured in position by mini-plates, thus enlarging the forehead anteriorly and thereby correcting the lateral SO recession, and hence, the SO bandeau is remodeled (Figure 3) [5].

Total calvarial reconstruction (TCVR)

A strip craniectomy of coronal suture from side to side is done, removing intact strips of bone up to the temporal region about 2.5 cm superior to the zygomatic arches. The bone overlying the sagittal sinus is disconnected bilaterally, about 2 cm from the mid-sagittal plane, leaving a 4 cm midline strip in situ. This midline strip is disconnected from the occipital bone just behind the lambdoid suture, about 5 cm above the superior nuchal line. This posterior disconnection is extended along both sides towards the skull base [5].

**Figure 4:**
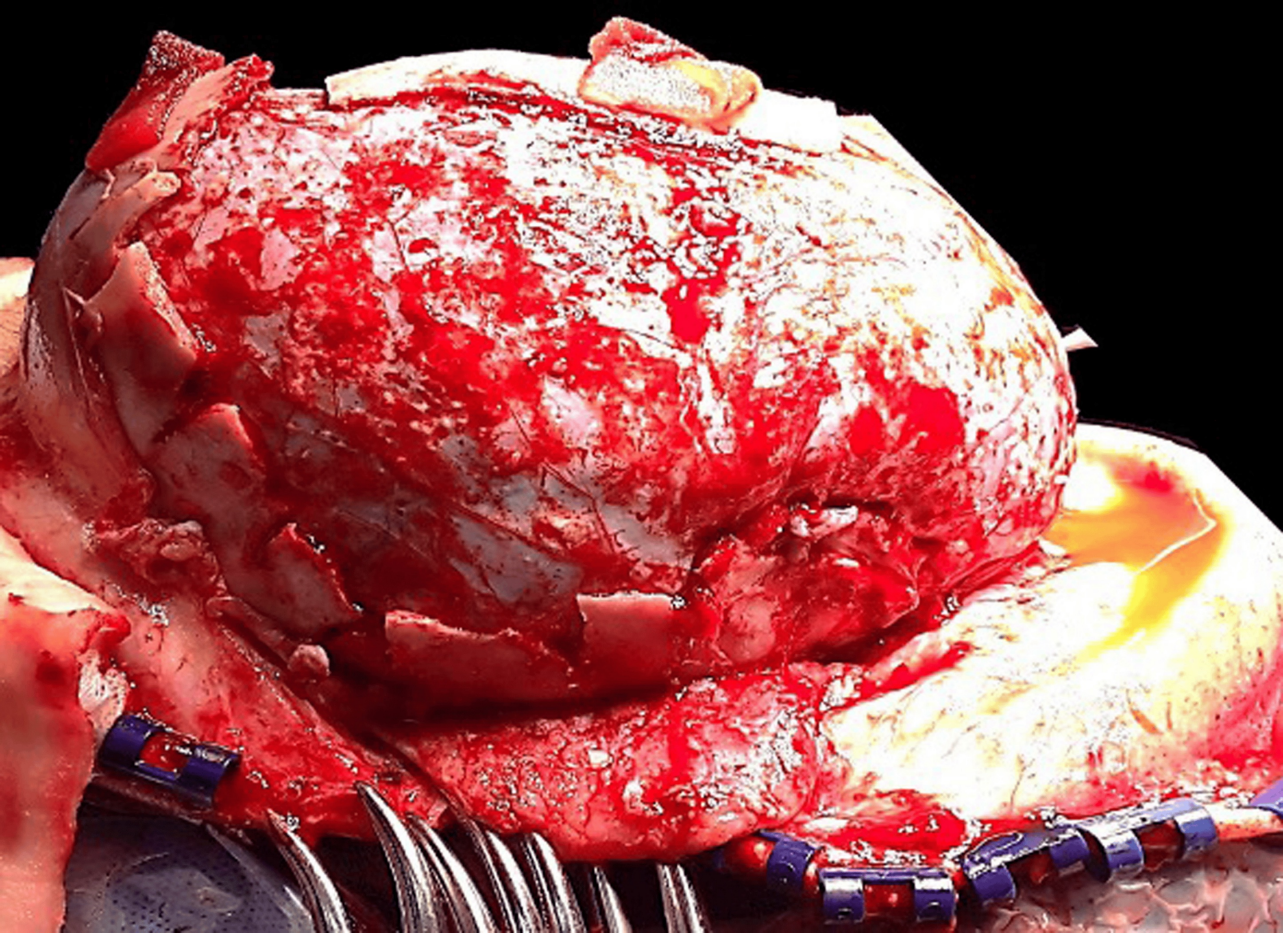
Out-fracturing of bone strip in TCVR TCVR - total calvarial reconstruction

**Figure 5:**
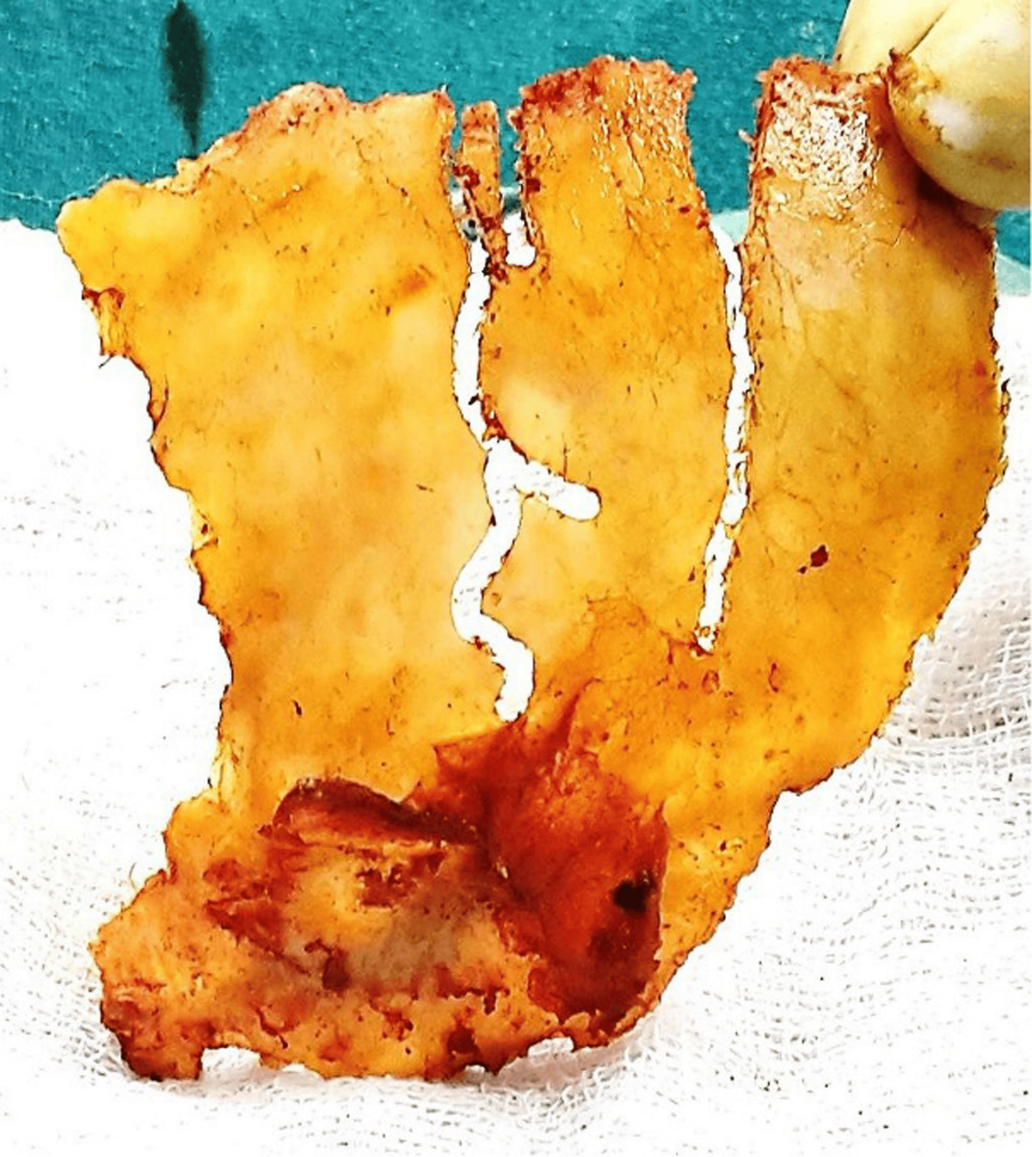
Flattening of the TPO flaps in TCVR TPO - temporo-parieto-occipital, TCVR - total calvarial reconstruction

**Figure 6:**
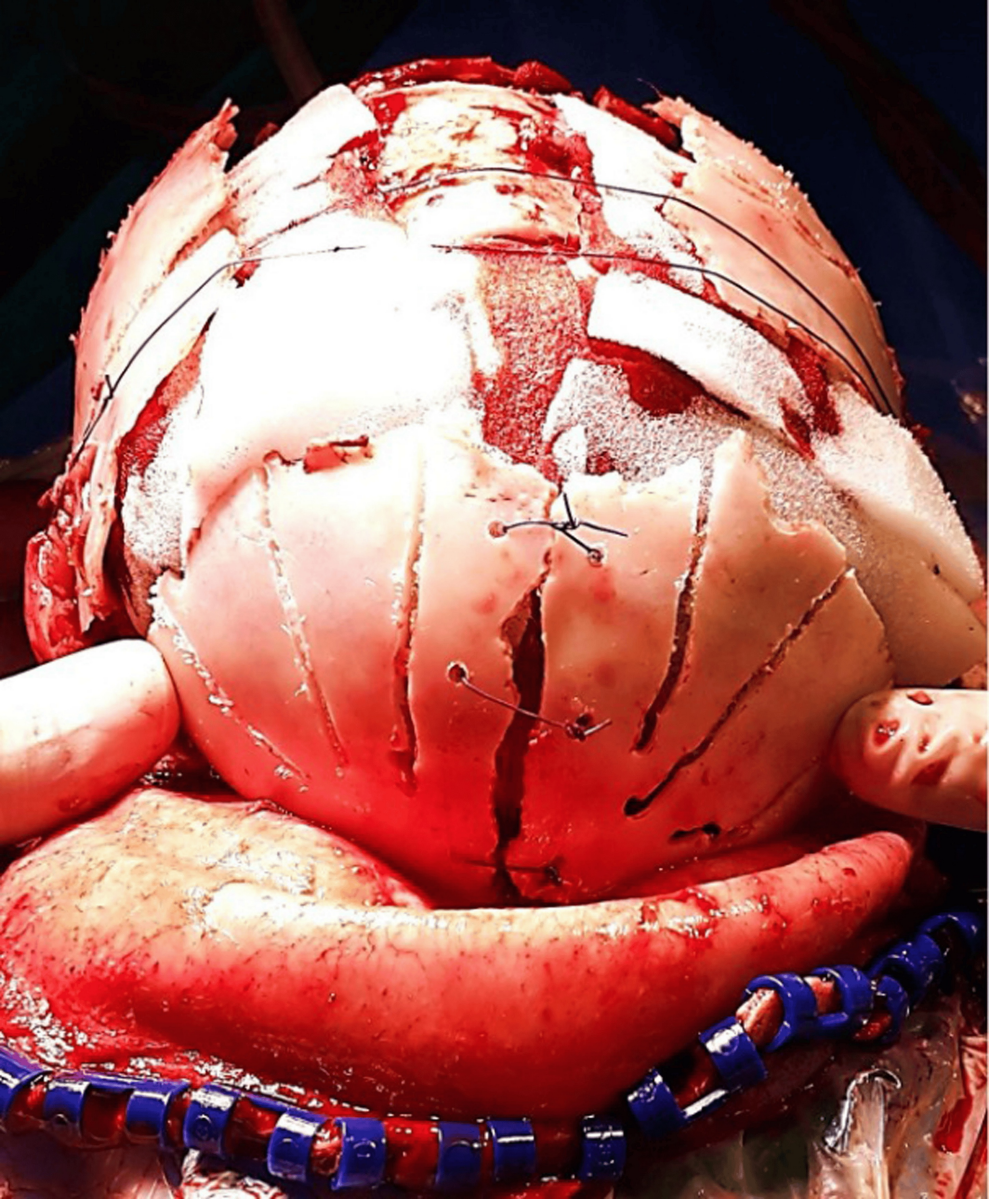
Inferior edges of TPO flaps tied loosely to inferior rim of bone laterally TPO - temporo-parieto-occipital

The temporo-parietal-occipital (TPO) flaps are removed from both sides, thus leaving 2.5 cm of bone towards the skull base in situ. An SO bandeau of about 2.5 cm is formed after the detachment of bilateral frontal bone flaps [5]. We fashion a BSO of the temporal base and occipital bone posteriorly with "out-fracturing" of bone fragments as low as possible. We perform this very cautiously by initially performing the BSO and then gripping the bone fragments with a duck-bill bone nibbler and out-fracturing each strip superior to the transverse sinus posteriorly and up to the temporal base laterally (Figure 4). The BSO of the removed TPO flaps is done to flatten them out, which helps in decreasing the curvature of bone flaps, which helps in cranial vault expansion (Figure 5) [5]. FBR with FOA is then carried out as described above and connected loosely with prolene sutures to remodeled TPO flaps, creating free floating single frontal flaps anteriorly and two TPO flaps posterolaterally on either side of the midline bone island left in-situ. This is done to protect the sagittal sinus and creates a triple bonnet configuration [5]. The inferior edges of TPO flaps are then tied loosely to the inferior rim of bone laterally (Figure 6).

Suturectomy (S)

A surgical cut is made directly over the affected suture, exposing its entire length. At both ends of fixed sutures, such as those crossing the superior sagittal sinus on each side in cases of metopic or sagittal craniosynostosis, small drill holes are created. The dura is gently separated from the bone above it. Using a craniotome, the bone is cut between the drill holes, removing the fused suture along with a narrow margin of normal bone. The incision is then closed in all layers [6].

Parent satisfaction score

To evaluate the perceived aesthetic outcome of surgery, a parent-reported satisfaction score was recorded for each patient. These results were recorded in the outpatient setting six months following the operation. The scale utilized ranges graded from 1-10: poor outcome (1-3), satisfied with outcome (4-7), and good outcome (8-10).

Surgical classification system

A surgical classification system was used and adapted for this study, as previously described by Sloan et al., which is shown in Table 1. We chose the Sloan Surgical Outcome Class because it is a validated scoring system that is applicable to all types of craniosynostosis and generates uniform, quantified data. This scoring system integrates parameters such as cranial deformity, surgical complexity, and postoperative results, providing a holistic assessment that is essential for managing diverse craniosynostosis cases. The operating surgeon utilized this classification system to assign an outcome report for each patient in this study.

Other methods considered were as follows. While three-dimensional (3D) photography offers high specificity and sensitivity for detecting craniosynostosis, it does not holistically assess surgical outcomes and was not considered to be cost-effective in this study [7]. The Cephalic index, which is traditionally used to evaluate craniosynostosis, is limited by high inter-observer variability and cannot reliably capture the 3D aspects of craniosynostosis [8]. Other standard methods, such as the Metopic Severity Score, tend to be specific to certain types of craniosynostosis and do not generalize well to our heterogenous dataset [9].

**Table 1:** Sloan's Surgical Outcome Class A lower number indicates a more favorable score [4]

Sloan's Surgical Outcome Class	
Class 1	Good to excellent correction, with no visible or palpable irregularity
Class 2	Good to excellent correction with palpable but not visible irregularity (e.g., a palpable but not visible surgical wire, plate, or bony irregularity), not requiring reoperation
Class 3	Good to excellent correction with visible irregularity (e.g., a visible prominence from a surgical wire or plate, or a visible bony spicule or defect that does not compromise the overall correction), not requiring reoperation
Class 4	Good to excellent correction with visible or palpable irregularity requiring reoperation (e.g., a surgical plate requiring removal)
Class 5	Compromised overall correction, but not severe enough to require reoperation (e.g., slight forehead asymmetry)
Class 6	Compromised overall correction requiring reoperation
Class 7	Compromised overall correction, believed to require reoperation by the surgeon, but family declines further surgery

Determination of head circumference

Head circumference was measured using manual tape just along the supra-orbital anteriorly and inion posteriorly. These measurements were made two weeks after the respective operations were performed.

Postoperative care and follow-up

After the operation, patients were kept in the Neurosurgery Intensive Care Unit (ICU) for 24 hours for monitoring, then shifted to the neurosurgery ward. Pain was managed with paracetamol suppositories. The follow-up duration of the study was six months in total.

Statistical analysis

Pre- and postoperative head circumferences (cm) were compared for all 26 patients using a paired t-test (p < 0.05). An unpaired t-test was performed to compare our recorded variable for all 26 patients to evaluate each of our recorded variable (age at diagnosis (months old), pre- and postoperative head circumference (cm), increase in head circumference post-intervention, preoperative hemoglobin level, transfusion requirement (mL), length of hospital stay (days), operative time (hours), parent satisfaction score, and Sloan's Surgical Outcome Class). Data analysis in this study was conducted using SPSS Statistics version 26.0 (IBM Inc., Armonk, New York) and Microsoft Excel version 16.84 (Microsoft, Redmond, Washington).

## Results

The clinical data of patients in group 1 and group 2 is shown in Table 2 and summarized in Table 3. The results from Table 3 are depicted in the Appendix. Out of 26 patients, 30.8% of patients were in group 1 and 69.2% of patients were in group 2. Though the patients in group 1 were younger (6.8±0.5 months) than patients in group 2 (12.7±2.3 months), the difference in age was not significant. Preoperative hemoglobin levels were comparable, but the difference in requirement of intra-op blood transfusion, operative time, and hospital stay time were significantly different, thus highlighting the distinction between less and more extensive procedures. The post-operative increase in head circumference was compared, and group 2 patients had a statistically significant increase (2.39±0.22 cm) compared to group 1 (0.74±0.08 cm). Sloan's Surgical Outcome Class and parent satisfaction score at six months between the two groups demonstrated a statistically significant difference with results favoring FBR with FOA or TCVR (group 2). Sloan's Surgical Outcome Class and parent satisfaction score consistently favor group 2 patients in terms of long-term surgical outcome class and better cosmetic acceptance by the parents.

**Table 2:** Parameters and Results of Patients Operated for Craniosynostosis Hgb - hemoglobin, S - suturectomy, FBR - frontal bone remodeling, FOA - fronto-orbital advancement, TCVR - total calvarial reconstruction, M - male, F - female

Case No. and craniosynostosis subtype	Year of surgery	Operation type	Group	Gender	Age (months)	Pre-operative Hgb (g/dL)	Transfusion required (mL)	Operative time (hours)	Hospital stay (days)	Parent Satisfaction Score (1-10)
1. Metopic	2005	S	1	M	8	10.4	0	2	4	5
2. Metopic	2009	FBR with FOA	2	F	12	12.5	0	4.3	5	9
3. Metopic	2010	FBR with FOA	2	M	14	11.6	120	4	7	9
4. Pansynostosis	2012	TCVR	2	M	48	10.7	200	6	7	10
5. Metopic	2012	FBR with FOA	2	M	8	14.5	0	3.5	8	10
6. Metopic	2013	FBR with FOA	2	M	6	13.4	100	4	7	10
7. Coronal unilateral	2013	S	1	F	5	12.8	0	2	4	4
8. Metopic	2014	FBR with FOA	2	M	11	13.0	70	4.8	4	9
9.Coronal bilateral	2014	S	1	F	7	11.2	150	2.3	5	5
10. Coronal bilateral	2015	FBR with FOA	2	M	8	10.7	230	3.5	5	10
11. Sagittal	2015	TCVR	2	M	12	10.5	250	4.3	8	9
12. Metopic	2016	FBR with FOA	2	M	9	11.5	100	3.6	5	9
13. Metopic	2016	FBR with FOA	2	F	10	10.6	80	3.8	4	8
14. Metopic	2016	S	1	M	8	11.0	0	2.2	4	5
15. Metopic	2017	FBR with FOA	2	F	12	12.2	0	4.4	5	8
16. Metopic	2017	FBR with FOA	2	M	8	10.8	0	4	5	9
17. Pansynostosis	2017	TCVR	2	M	24	11.0	200	4.3	6	9
18. Metopic	2018	FBR with FOA	2	M	8	11.2	0	3.2	6	10
19. Metopic	2018	S	1	M	6	11.0	0	3.4	5	4
20. Coronal unilateral	2018	S	1	F	8	9.8	0	2.3	5	6
21. Metopic	2019	FBR with FOA	2	M	9	10.0	0	2.0	4	9
22. Coronal bilateral	2019	FBR with FOA	2	F	8	11.0	50	4.1	4	8
23. Coronal bilateral	2019	S	1	M	8	9.6	0	2.3	5	6
24. Sagittal	2020	TCVR	2	M	12	10.7	120	5	7	9
25. Metopic	2020	FBR with FOA	2	M	9	11.0	50	4	5	9
26. Metopic	2021	S	1	F	5	12.3	0	1.8	4	6
Mean		-		18M; 8F	10.9	11.3	66	3.5	5.3	7.9

**Table 3:** Comparison of Patients Operated for Synostosis (S)*: Statistically significant (p-value <0.05), (NS) - not significant, SEM - standard error of the mean, FBR - frontal bone remodeling, FOA - fronto-orbital advancement, TCVR - total calvarial reconstruction

Variables	Group 1 (Suturectomy)	Group 2 (FBR w/ FOA, TCVR)	p-value
	Mean ± SEM	Mean ± SEM	
Age at diagnosis (months)	6.9 ± 0.5	12.7 ± 2.3	0.108 (NS)
Pre-operative hemoglobin (g/dL)	11.01 ± 0.39	11.49 ± 0.28	0.339 (NS)
Transfusion requirement (mL)	18.8 ± 18.8	87.2 ± 20.1	0.048 (S)*
Operative time (hours)	2.29 ± 0.17	4.04 ± 0.19	<0.001 (S)*
Hospital stay (days)	4.5 ± 0.2	5.7 ± 0.3	0.005 (S)*
Pre-op head circumference (cm)	41.32 ± 0.32	43.53 ± 0.47	0.007 (S)*
Post-op head circumference (cm)	42.06 ± 0.32	45.92 ± 0.60	<0.001 (S)^*^
Increase in head circumference post-surgery (cm)	0.74 ± 0.08	2.39 ± 0.22	<0.001 (S)^*^
Surgical outcome class	5.4 ± 0.5	1.2 ± 0.1	<0.001 (S)^*^
Parent satisfaction score	5.1 ± 0.3	9.1 ± 0.2	<0.001 (S)^*^

## Discussion

Craniosynostosis, a heterogeneous group of disorders, is one of the most common malformations in children presenting in the first year of life [10]. Premature closure of sutures results in cranial deformity and compromised intracranial volumes, resulting in raised ICP and secondary brain damage [11,12]. With a reported incidence of 1:4000-1:6000, a recent epidemiological study has confirmed an increasing incidence of craniosynostosis [13-15].

Sagittal synostosis followed by coronal synostosis is the most common type of craniosynostosis reported in literature [4,16]. Morrison et al., in a pooled cohort of both children and adults, reported unicoronal suture as the most affected suture [17]. A similar observation was made by Abdallah et al. [18]. In another study performed in the Middle East, metopic synostosis was the most common type of craniosynostosis [19]. In our study of 26 patients, metopic synostosis was disproportionately the largest group (61.5%).

Surgical techniques to correct craniosynostosis can be divided into two groups: procedures that involve the removal of affected sutures (suturectomies, endoscopic, or open) and calvarial vault remodeling/reconstructions with or without front-orbital advancement [20]. The advantages of later include controlled remodeling of different areas of vault simultaneously and achieving normal cranial indices when compared to suturectomies [21,22]. Disadvantages include a more extensive surgical procedure with greater technical difficulty, increased blood loss, increased transfusion rates and longer hospital stay, with increased potential adverse effects [23,24] An standardized grading system assessing cranial vascular injury, such as the one outlined in Koenig et al., may play an important role in guiding treatment [25].

Craniosynostoses are generally not recognized or referred to at a late stage [26]. This holds more true for developing countries like India where patients present late due to lack of medical education, poverty, local beliefs, and paucity of medical facilities [3]. In developed countries such as the United States, Black and Hispanic children were more likely to present later and undergo open calvarial vault reconstruction. This contrasts with Caucasian children, who present early and undergo endoscopic suturectomies [27]. Early referral to a specialist center well before the age of six months ensures the option of minimally invasive surgical options, as compared to late referrals beyond six months of age when open corrections are preferably done [26]. Addressing these issues requires a combination of surgical outreach programs, targeted healthcare policies, and training opportunities for physicians to expand care into rural and underserved areas, as discussed by Koenig et al. [28].

The exponential growth of the brain is largely complete at three years of age [29]. The optimal timing and technique for reconstruction are debated, but surgical intervention is usually performed within one year of life [30-32]. A survey by Synostosis Research Group found a substantial variation in the choice of surgical procedure and technique. Their procedure of choice for children less than three months of age is suturectomies, and for patients over six years of age is open vault surgery [33]. CVR involves extensive craniotomies, removing involved sutures, remodeling of bones using plates and sutures, and repositioning of manipulated bone segments in specific areas [6]. Despite advances in the management of craniosynostosis, open surgical procedures are still crucial for the treatment of infants more than six months of age and, with proper precautions, can be performed with minimal complications, low recurrence rates, and satisfactory cosmetic outcomes [19]. Similarly, Fearon et al. [34] found shorter length of stay (2.5 days), lower complication and re-operation rates of 0.4% and 2.0%, respectively, for CVRs.

In developing countries like India, where children present late for treatment, we offered open surgical procedures to all patients. We operated on 18 of our patients using a technique or its variation previously described by Raswan et al. [5]. Due to the costliness of absorbable plates, we used prolene sutures to tie up the free bone edges to create free floating flaps, which were continuously remodeled by the centrifugal forces created by the growth of the brain on the dura. By keeping the central part of the sagittal sinus covered by a floating central island of bone, the risk of sudden and torrential loss of blood is reduced. Titanium plates were used for the reconstruction of the fronto-orbital bandeau, thus controlling hypotelorism and supraorbital ridge shape. Eight patients underwent simple suturectomies in the earlier part of our experience. Our results show that open surgeries can be performed safely in experienced hands with good surgical outcomes.

Except for a single case in 2005, where only suturectomy was offered, all parents were offered both suturectomies and more extensive procedures as treatment options. The risks and outcomes for both types of procedures were explained in detail. The main criteria for performing one surgery over another was ultimately decided by parental choice. This was based on parental perception of the risks and benefits of each type of operation.

Extensive surgeries like CVR in our study have shown better results than simple suturectomies in terms of Sloan Surgical Outcome class and parent satisfaction outcome scores, albeit at significantly increased chance of intraoperative bleeding, transfusion rates, and longer hospital stay without any significant increase in morbidity or mortality. Schulz et al. [35] believe that the subjective perception of the morphometric results as perceived by the parents might be the most important outcome parameters. We used parent satisfaction scores, which showed significantly higher scores for the extensive surgery group. To evaluate cosmetic outcome and need for repeat surgery, the classification described by Sloan et al. was used [4]. Classes 1 to 4 are excellent to good overall correction while classes 5 to 7 are a compromised correction, requiring repeat surgery [4].

In comparison to less extensive procedures, the more extensive procedures reported statistically significant better outcomes in terms of subjective perception of morphometric results as perceived by parents (9.1±0.2 vs 5.1±0.3) and cosmetic results as measured using Sloan's Surgical Outcome Class (1.2±0.1 vs 5.4±0.5). Since the Sloan Surgical Outcome Class determines the outcome in terms of whether revision surgery is required or not, no patient with the Sloan Surgical Outcome Class of 1-5 required surgery. Patients with Sloan classes 6 and 7 were offered revision surgery, but the parents declined further intervention.

The overall assessment of pre vs. postoperative head circumference revealed a strong significant mean improvement from 42.85 cm to 44.73 cm (p < 0.001) and, therefore, an indirect increase in intracranial volume, as shown in Table 4. The difference in the overall increase in head circumference was also statistically significant, favoring the extensive surgery group. It should be noted that while more extensive procedures such as FBR with FOA and TCVR demonstrated better outcomes, parental preference during pre-operative discussions played a key role in the continued use of less extensive procedures.

**Table 4:** Preoperative and postoperative head circumferences of children with non-syndromic craniosynostosis S  - statistically significant (p-value < 0.05), SEM - standard error of the mean

Case No.	Pre-operative (cm)	Pre-operative (percentile)	Post-operative (cm)	Post-operative (percentile)	Increase in head circumference (cm)
1S	41.3	25	42.2	50	0.9
2	41.3	15	43.4	75	2.1
3	43.3	50	46.0	98	2.7
4	47.5	3	53.0	97	5.5
5	43.0	40	45.3	95	2.3
6	40.4	15	43.3	94	2.9
7S	40.5	25	41.3	50	0.8
8	43.1	25	44.8	75	1.7
9S	40.1	3	40.9	5	0.8
10	45.8	85	47.6	99	1.8
11	46.0	50	49.0	99	3.0
12	44.6	50	45.8	75	1.2
13	43.3	25	44.9	75	1.6
14S	41.7	1	42.2	3	0.5
15	41.3	1	43.8	25	2.5
16	43.0	15	44.6	50	1.6
17	45.7	3	48.4	50	2.7
18	42.1	15	44.2	75	2.1
19S	42.0	70	43.1	90	1.1
20S	42.8	25	43.2	50	0.4
21	40.4	15	42.4	75	2.0
22	44.3	75	46.6	99	2.3
23S	41.6	1	42.3	3	0.7
24	45.0	25	48.0	95	3.0
25	43.4	15	45.5	75	2.1
26S	40.6	25	41.5	50	0.9
Mean ± SEM (cm)	42.85 ± 0.39		44.73 ± 0.55		

This study has several limitations that should be acknowledged. The small sample size may limit the statistical power and restrict the generalizability of the findings to a broader population. Additionally, the retrospective nature of the study introduces potential biases, such as selection bias and missing data, which may affect the robustness of the conclusions. As a single-center study conducted at a tertiary care hospital in India, the findings may not be fully applicable to other healthcare settings, particularly those with different patient demographics or healthcare infrastructures. Procedures such as helmet therapy and endoscopic suturectomy were excluded from this study based on cost, as the majority of patients came from underserved backgrounds. Evaluating these procedures may be beneficial in countries with the financial means to provide them and are an excellent area for future study.

The use of parental satisfaction scores, while essential, is prone to cultural and personal biases. As the parental satisfaction scores were gathered in an outpatient setting, these results are prone to interviewer bias. Objective techniques such as 3D photography should be used in future studies to provide a more accurate and objective assessment of craniosynostosis [7].

Additionally, the more extensive procedures were performed by a single surgeon, while the less extensive procedures were performed by a different surgeon, potentially introducing bias based on individual skill. Finally, the time course over which the study took place may also play a role in the results, as technique or individual skill may improve over time. While Table 2 suggests that the year of surgery did not play a large role, future studies should ideally take place over a shorter time span to account for these variations. Long-term follow-up should be expanded to include developmental, neurological, and cosmetic outcomes at regular intervals. These limitations highlight the need for a larger, multi-center trial and prospective randomized controlled trials performed to validate our results and enhance their applicability across diverse populations.

## Conclusions

We have reviewed our 16-year experience with the surgical management of craniosynostosis at a single center. The results show that craniosynostosis can be successfully corrected with low morbidity. With experience, our management of this complex abnormality has evolved from simple suturectomies to more complex calvarial reconstructions. Although procedures involving calvarial reconstructions are more extensive, have a greater duration, and are associated with more blood loss, they have better long-term cosmetic and functional results with acceptable mortality and morbidity. In developing countries like India, where children present late and sometimes resorbable plates, internal distractors and helmets are either unaffordable or unavailable. The simple technique described by us, creating free flaps joined together with loosely tied prolene sutures, provides excellent surgical outcomes. We hope that this study will contribute to our evolving understanding and treatment of craniosynostosis.

## Figures and Tables

**Figure 7:**
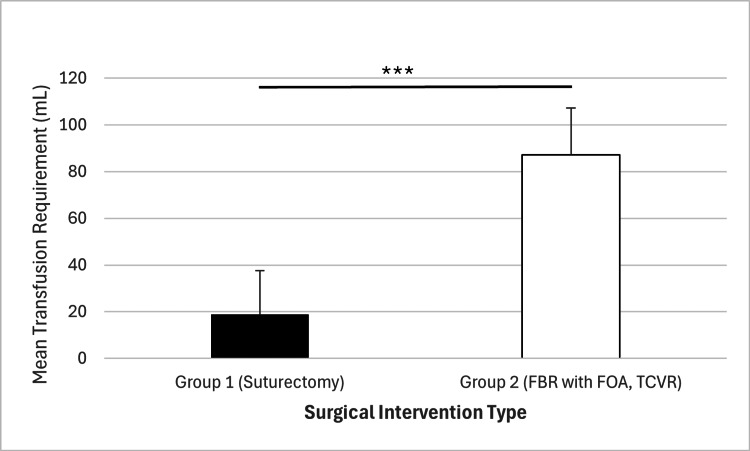
Transfusion requirement FBR - frontal bone remodeling, FOA - fronto-orbital advancement, TCVR - total calvarial reconstruction Data is shown as mean ± standard error of sample mean. A triple asterisk indicates a statistically significant difference with a p-value ≤ 0.001.

**Figure 8:**
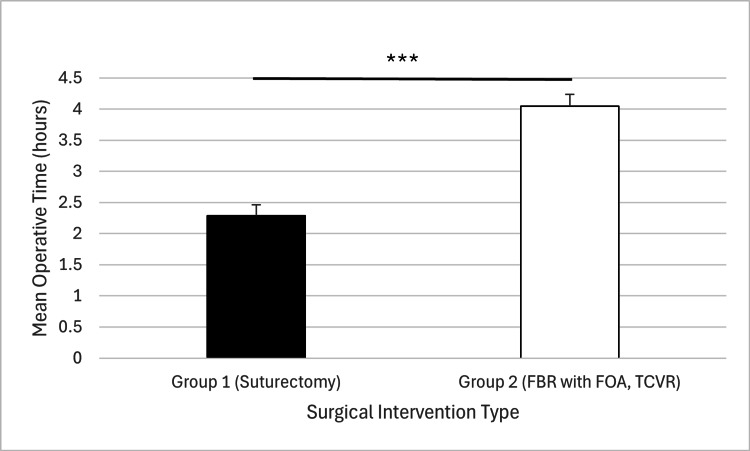
Operative time FBR - frontal bone remodeling, FOA - fronto-orbital advancement, TCVR - total calvarial reconstruction Data is shown as mean ± standard error of sample mean. A triple asterisk indicates a statistically significant difference with a p-value ≤ 0.001.

**Figure 9:**
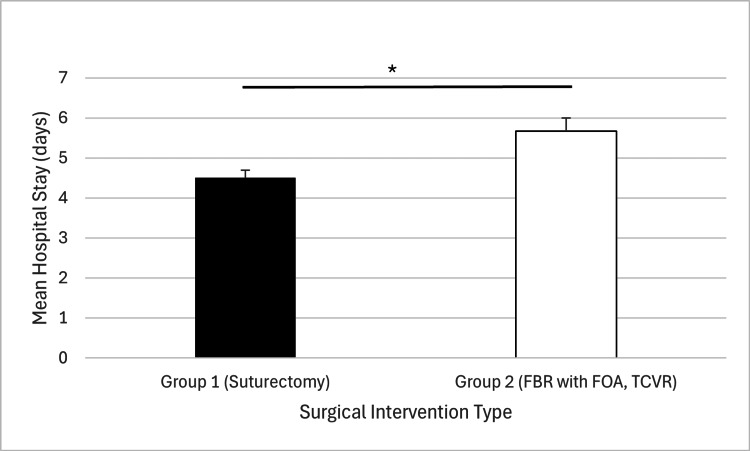
Hospital stay time FBR - frontal bone remodeling, FOA - fronto-orbital advancement, TCVR - total calvarial reconstruction Data is shown as mean ± standard error of sample mean. A single asterisk indicates a statistically significant difference with a p-value ≤ 0.05.

**Figure 10:**
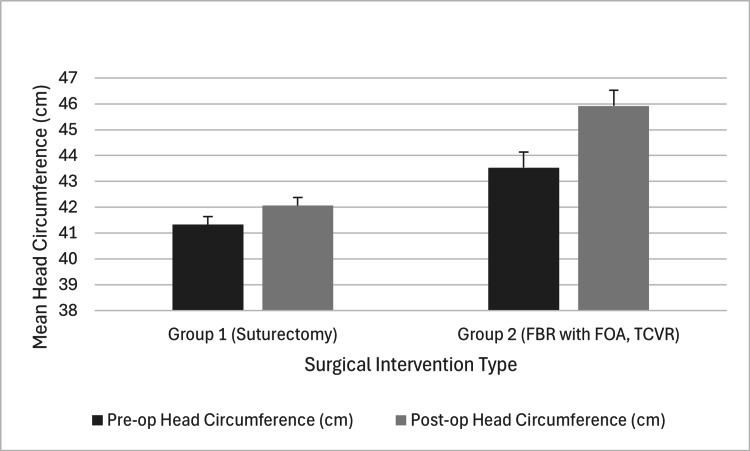
Comparison of pre-and postoperative head circumference FBR - frontal bone remodeling, FOA - fronto-orbital advancement, TCVR - total calvarial reconstruction Data is shown as mean ± standard error of sample mean.

**Figure 11:**
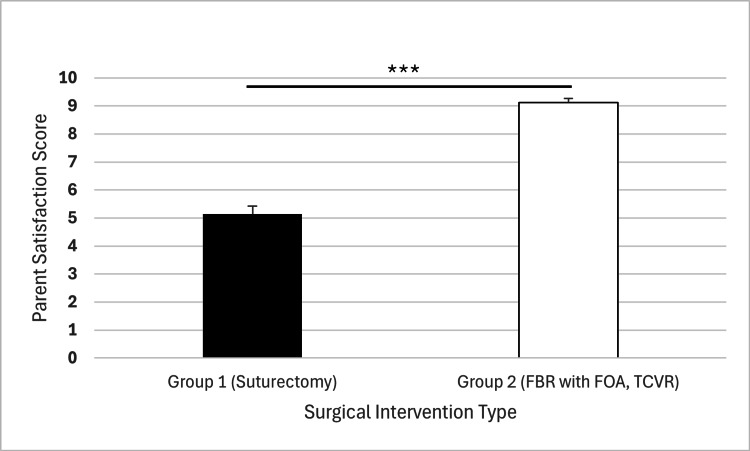
Parent Satisfaction Score FBR - frontal bone remodeling, FOA - fronto-orbital advancement, TCVR - total calvarial reconstruction A higher number indicates greater satisfaction. Data is shown as mean ± standard error of sample mean. A triple asterisk indicates a statistically significant difference with a p-value ≤ 0.001.

**Figure 12:**
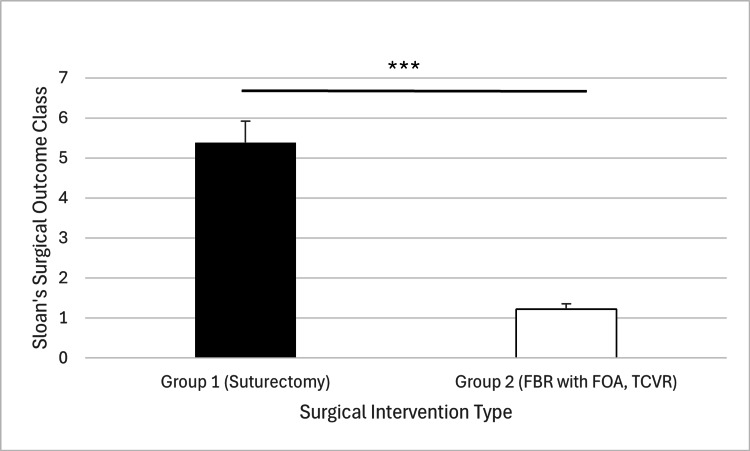
Sloan's Surgical Outcome Class FBR - frontal bone remodeling, FOA - fronto-orbital advancement, TCVR - total calvarial reconstruction A lower number indicates a more favorable outcome [4]. Data is shown as mean ± standard error of sample mean. A triple asterisk indicates a statistically significant difference with a p-value ≤ 0.001.

## Appendices

As depicted in Figures 7-12, group 1 (suturectomy) showed clinically significant differences with group 2 (FBR with FOA, TCVR) as corrective surgical procedures for metopic synostosis. The original data can be seen in Table 3.
